# The Absolute Configuration of Salicortin, HCH-Salicortin and Tremulacin from *Populus trichocarpa × deltoides* Beaupré

**DOI:** 10.3390/molecules20045566

**Published:** 2015-03-30

**Authors:** Felix Feistel, Christian Paetz, Sybille Lorenz, Bernd Schneider

**Affiliations:** Max Planck Institute for Chemical Ecology, Hans-Knöll-Straße 8, Jena 07745, Germany; E-Mails: ffeistel@ice.mpg.de (F.F.); cpaetz@ice.mpg.de (C.P.); lorenz@ice.mpg.de (S.L.)

**Keywords:** salicortin, HCH-salicortin, tremulacin, idescarpin, salicinoids, absolute configuration

## Abstract

The absolute configuration of salicortin, HCH-salicortin and tremulacin, isolated from leaves of *Populus trichocarpa × deltoides* Beaupré, was determined by comparing spectroscopic data of these compounds with those of idescarpin, isolated from leaves of *Idesia polycarpa*. All compounds were characterized by nuclear magnetic resonance spectroscopy, high-resolution mass spectrometry, and circular dichroism spectroscopy. It was found that the hydroxy cyclohexenonoyl (HCH) moiety in all compounds is (*S*)-configured. In addition, it was shown that leaves of *Idesia polycarpa* contain high amounts of (−)-idescarpin (1.1%, based on dry weight).

## 1. Introduction

**Figure 1:**
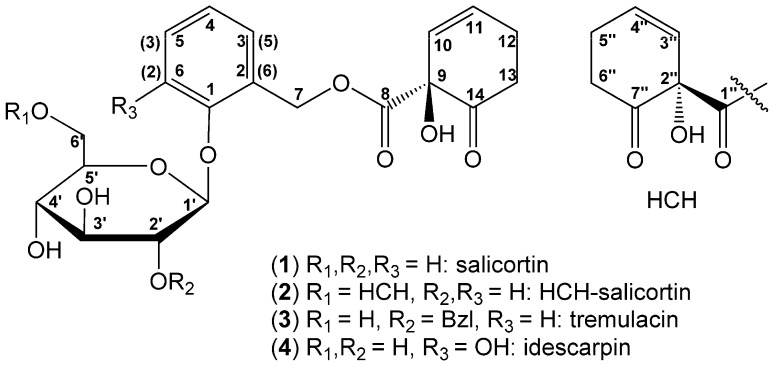
Structures of salicortin (**1**); HCH-salicortin (**2**); tremulacin (**3**) and idescarpin (**4**). Note the different numbering for idescarpin are in parentheses.

The salicinoids salicortin (**1**), HCH-salicortin (**2**) and tremulacin (**3**) (Figure 1) are phenolic secondary metabolites occurring in the Salicaceae family; they serve the plants as defense compounds with feeding deterrent and antifungal properties [1,2,3,4,5]. The mechanism underlying the biological activity is thought to be based on degradation, occurring either enzymatically or at pH > 7, and resulting in the production of saligenin, which is toxic to insects [5]. It has been shown that the hydroxy cyclohexenonoyl (HCH) moiety is transformed into pyrocatechol, which subsequently interacts with proteins responsible for digestion by leaf-feeding organisms [6]. It has also been reported that salicinoids show anti-adipogenic activity [7,8,9], melanine biosynthesis inhibition [10], and nitric oxide production [11].

Questions remain, however, about salicortin (**1**), which is isolated and was described many years ago [12], its biosynthesis [13], and the absolute configuration of its structure. Recently, the absolute configuration of the structurally very similar salicinoid (−)-idescarpin (**4**) (Figure 1), isolated from fruits of *Idesia polycarpa* [14], was elucidated using single-crystal X-ray diffraction studies. Here, we report on the absolute configuration of salicortin (**1**), HCH-salicortin (**2**) and tremulacin (**3**) isolated from leaves of *Populus trichocarpa* × *deltoides* Beaupré by comparing spectroscopic data from these compounds with those of idescarpin (**4**).

## 2. Results and Discussion

**Figure 2:**
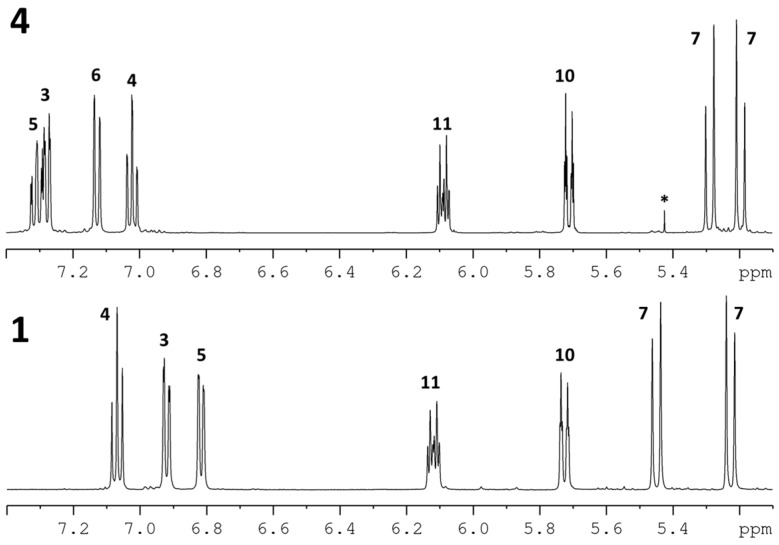
Partial ^1^H-NMR spectra (500 MHz) of isolated salicortin (**1**) and idescarpin (**4**) in MeCN-*d*_3_ with position numbering according to Figure 1. The signal marked with * represents an unidentified impurity.

**Figure 3:**
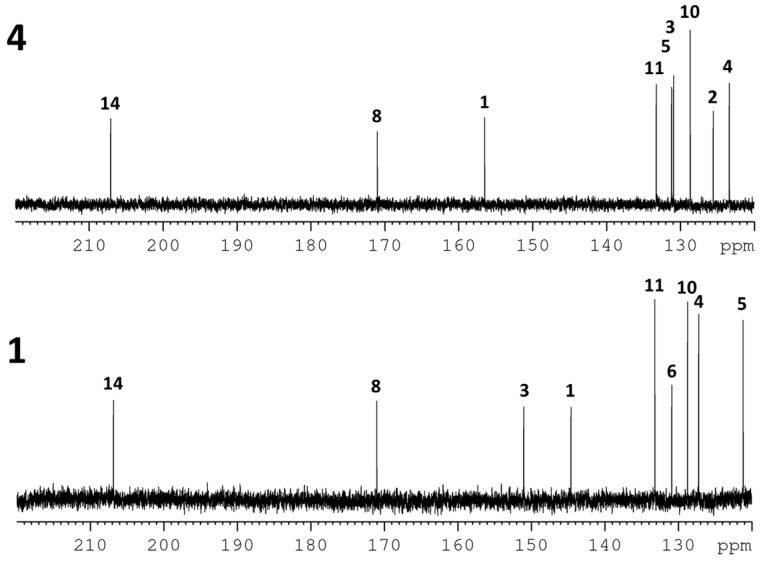
Partial ^13^C-NMR spectra (125 MHz) of isolated salicortin (**1**) and idescarpin (**4**) in MeCN-*d*_3_ with position numbering according to Figure 1.

**Figure 4:**
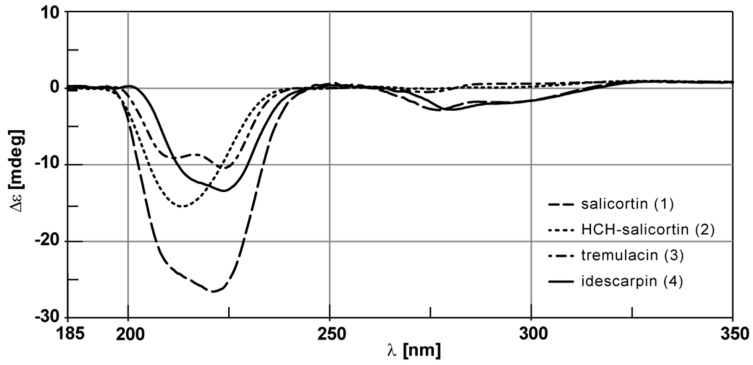
Superimposed CD spectra of isolated salicortin (**1**), HCH-salicortin (**2**), tremulacin (**3**) and idescarpin (**4**).

Salicortin (**1**) was isolated from *Populus trichocarpa* × *deltoides* Beaupré leaves in 0.3% yield. HCH-salicortin (**2**) and tremulacin (**3**) were isolated in yields of 0.1% and 0.2%, respectively. Idescarpin (**4**) was isolated from leaves of *Idesia polycarpa* in a similarly high yield of 1.1% (all yields based on dry leaf weight). The methods described in the experimental section and in the supplementary information were used for separation. The structures of all compounds were confirmed by nuclear magnetic resonance (NMR) spectroscopy (Table 1 and supplementary information) and high-resolution mass spectrometry (HRMS) (see supplementary information). Recorded spectral data were consistent with the structures and in accordance with reported data [14,15,16,17]. The signals of neither ^1^H- nor ^13^C-NMR spectra show additional splitting or broadening, thus suggesting that all compounds are present as single diastereomers (Figure 2 and Figure 3). The specific optical rotation of salicortin (**1**) was initially reported to be ${\left[\text{α}\right]}_{\text{D}}^{20}$ = −164.2 (c 1.38, H_2_O) [12], but the absolute configuration has not yet been determined. The specific rotation of (−)-idescarpin (**4**) has been reported to be ${\left[\text{α}\right]}_{\text{D}}^{25}$ = −156.6 (c 1.0; tetrahydrofuran) [14]. For tremulacin (**3**), a specific rotation of ${\left[\text{α}\right]}_{\text{D}}^{25}$ = −134.7 (c 0.59; MeOH) has been reported [17]. No optical rotation data for **2** have been found in the literature. To determine the absolute configuration, the specific optical rotation and the circular dichroism (CD) spectra of all isolated compounds were measured and compared. The specific optical rotation of isolated salicortin (**1**) was determined to be ${\left[\text{α}\right]}_{\text{D}}^{22}$ = −118.6 (c 0.65; H_2_O) and ${\left[\text{α}\right]}_{\text{D}}^{22}$ = −123.9 (c 0.72, MeOH). For (−)-idescarpin (**4**), we determined a specific optical rotation of ${\left[\text{α}\right]}_{\text{D}}^{22}$ = −57.3 (c 0.73, MeOH). The negative values point to the same configuration at C-9 of **1** and **2**. Due to limited availability of the compounds, the specific optical rotation of compounds **2** and **3** was not determined. However, the CD spectra of all compounds show high similarities (Figure 4 and Supplementary Information). The following molar circular dichroism values Δε have been determined: Salicortin (**1**) Δε= −26.7 mdeg (λ_max_ = 221 nm, c = 1.66 mM); HCH-salicortin (**2**) Δε= −15.5 mdeg (λ_max_ = 213 nm, c = 1.19 mM); tremulacin (**3**) Δε = −9.2 mdeg (λ_max_ = 211 nm, c = 1.44 mM) and Δε = −10.5 mdeg (λ_max_ = 239 nm, c = 1.44 mM); idescarpin (**4**) Δε = −13.4 mdeg (λ_max_ = 224 nm, c = 1.61 mM). We conclude that the configuration at C-9 (and C-2" in HCH-salicortin) in all isolated compounds is identical and thus (*S*)-configured.

**Table 1:** ^1^H and ^13^C-NMR data of salicinoids in MeCN-*d*_3_. Salicortin (**1**) and idescarpin (**4**) and were measured at frequencies of 500 MHz for ^1^H and 125 MHz for ^13^C-NMR. HCH-salicortin (**3**) and tremulacin (**2**) were measured at 700 MHz and their ^13^C-NMR data were obtained from Heteronuclear Single Quantum Coherence (HSQC) and Heteronuclear Multiple Bond Correlation (HMBC) spectra.

Pos.	Salicortin (1)		Tremulacin (2)		HCH-salicortin (3)		Idescarpin (4) ^(1)^	
	δ_H_, mult., *J* (Hz)	δ_C_	δ_H_, mult., *J* (Hz)	δ_C_	δ_H_, mult., *J* (Hz)	δ_C_	δ_H_, mult., *J* (Hz)	δ_C_
1		156.3		155.8		156.3		144.4
2		125.4		125.6		126.0		150.9
3	7.30, *dd*, 7.5, 1.0	130.7	7.20, *d*, 7.6	130.0	7.35, *d*, 7.5	130.6	6.92, *dd*, 7.6, 1.2	118.3
4	7.05, *ddd*, 7.5, 7.5, 1.0	123.2	7.03, *dd*, 7.6, 7.6	123.6	7.11, *dd*, 7.5, 7.5	123.2	7.07, *dd*, 7.6, 7.6	127.0
5	7.33, *ddd*, 7.5, 7.5, 1.0	131.0	7.31, *dd*, 7.6, 7.6	130.7	7.41, *dd*, 7.5, 7.5	131.0	6.82, *dd*, 7.6, 1.2	121.0
6	7.15, *dd*, 7.5, 1.0	116.1	7.17, *d*, 7.6	116.2	7.13, *d*, 7.5	116.0		130.7
7a	5.30, *d*, 12.3	64.2	5.02, *d*, 12.5	63.5	5.30, *d*, 12.3	64.0	5.45, *d*, 12.6	64.5
7b	5.26, *d*, 12.3		4.78, *d*, 12.5		5.26, *d*, 12.3		5.23, *d*, 12.6	
8		170.8		170.4		170.3		170.9
9		78.9		78.7		78.7		79.0
10	5.74, *ddd*, 9.9, 1.8, 1.8	128.5	5.67, *d*, 9.8	128.4	5.69, *ddd*, 9.8, 1.6, 1.6	128.3	5.73, *ddd*, 9.9, 2.0, 2.0	128.6
11	6.11, *ddd*, 9.9, 3.4, 3.4	133.1	6.11, *ddd*, 9.8, 4.2, 4.2	132.9	6.09, *ddd*, 9.8, 3.7, 3.7	132.8	6.12, *ddd*, 9.9, 3.5, 3.5	133.0
12a	2.61, *m/*2.49, *m*	27.2	2.63, *m/*2.50, *m*	27.2	2.61, *m/*2.50, *m*	27.1	2.62, *m/*2.52, *m*	27.2
13a	2.85, *m/*2.52, *m*	36.2	2.83, *m/*2.50, *m*	36.0	2.83, *m/*2.52, *m*	36.0	2.90, *m/*2.54, *m*	36.2
14		206.9		206.8		206.7		206.7
1'	4.92, *d*, 7.5	101.6	5.25, *d*, 8.0	99.9	4.93, *d*, 7.8	101.5	4.57, *d*, 7.7	106.7
2'	3.42, *dd,* 9.0, 7.5	74.3	5.16, *d*, 8.0, 9.5	74.9	3.38, *dd*, 9.5, 7.8	74.0	3.45, *dd*, 8.8, 7.7	74.8
3'	3.46, *dd*, 9.0, 9.0	77.2	3.78, *m*	75.2	3.42, *dd*, 9.5, 9.5	76.9	3.39, *dd*, 8.8, 8.8	77.0
4'	3.39, *dd*, 9.0, 9.0	70.8	3.54, *m*	71.0	3.30, *dd*, 9.5, 9.5	70.6	3.34, *dd*, 8.8, 8.8	70.6
5'	3.42, *ddd*, 9.0, 5.4, 1.8	77.1	3.54, *m*	77.7	3.68, *ddd*, 9.5, 6.5, 2.0	74.7	3.29, *ddd*, 8.8, 5.2, 2.4	77.0
6'a	3.77, *dd*, 12.0, 1.8	62.2	3.84, *m*	62.1	4.53, *dd*, 12.0, 2.0	65.7	3.75, *dd*, 12.0, 2.4	62.1
6'b	3.61, *dd*, 12.0, 5.4		3.69, *m*		4.24, *dd*, 12.0, 6.5		3.62, *dd*, 12.0, 5.2	
1''				166.3		170.7		
2''				130.3		78.7		
3''			8.05, *d*, 7.6	130.3	5.74, *ddd*, 9.8, 1.7, 1.7	128.3		
4''			7.50, *dd*, 7.6, 7.6	129.4	6.12, *ddd*, 9.8, 3.5, 3.5	132.9		
5''			7.63, *dd*, 7.6, 7.6	134.2	2.61, *m/*2.50, *m*	27.1		
6''			7.50, *dd*, 7.6, 7.6	129.4	2.83, *m/*2.52, *m*	36.0		
7''			8.05, *d*, 7.6	130.3		207.1		

^(1)^: Please note different numbering of the aromatic ring (positions 2, 3, 5, and 6) in idescarpin (**4**) according to Figure 1 and Supplementary Figures S5.6.

## 3. Experimental Section

### 3.1. General Information

Solvents used for extraction and chromatographic separation were purchased from Carl Roth GmbH, Karlsruhe, Germany and VWR International GmbH, Darmstadt, Germany, and used without further purification. Water used for HPLC was obtained from a Milli-Q Synthesis A10 purifier (Merck KGaA, Darmstadt, Germany). HR-X SPE cartridges (500 mg sorbent/6 mL volume) were purchased from Macherey-Nagel, Düren, Germany. Sephadex LH-20 and polyamide colums DPA-6S were purchased from Sigma-Aldrich GmbH, Schnelldorf, Germany. Separation on Sephadex LH-20 was performed in column chromatography mode using MeOH as a solvent. HPLC separations were carried out on an Agilent 1100 HPLC system, consisting of a degasser, binary pump, autosampler and DAD detector (Agilent Technologies GmbH, Böblingen, Germany). The column outlet was connected to an Advantec CHF122SB fraction collector (Jasco GmbH, Gross-Umstadt, Germany) triggered by a relay board from the Agilent 1100. An Isis RP-18e column (250 × 4.6 mm, 5 µm particle size) from Macherey-Nagel, Düren, Germany, was used for all separations. HPLC gradients are given in the supplementary information. Fractions were evaporated using a Genevac HT-4X vacuum centrifuge (Genevac Ltd., Ipswich, UK). HRMS data were measured via direct injection on a LTQ Orbitrap XL mass spectrometer in positive ionization mode (Fischer Scientific GmbH, Schwerte, Germany). NMR spectra were recorded on a Bruker Avance 500 spectrometer equipped with a cryoplatform and a 5 mm TCI cryoprobe and on a Bruker Avance III HD spectrometer, equipped with a cryoplatform and a 1.7 mm TCI micro-cryoprobe (Bruker Biospin GmbH, Rheinstetten, Germany). NMR tubes of 5 mm and 1.7 mm outer diameter, respectively, were used for measurements. All NMR spectra were recorded using MeCN-*d*_3_ as solvent. Chemical shifts were referenced to the residual solvent peaks at δ_H_ 1.94 and δ_C_ 1.32. Data acquisition and processing were accomplished using TopSpin 2.1 and TopSpin 3.2, respectively. Standard pulse programs as implemented in TopSpin were used for data acquisition. Specific optical rotation was measured on a Jasco P-1030 polarimeter, CD spectra were recorded on a Jasco J-810 spectropolarimeter (Jasco GmbH, Gross-Umstadt, Germany). All compounds were measured in MeOH using a quartz cuvette of 1 mm width. Plants of *Populus trichocarpa* *× deltoides* Beaupré were raised in the greenhouse facilities of the Max Planck Institute for Chemical Ecology in Jena, Germany. The *Idesia polycarpa* leaf specimen was taken from a tree growing in the Botanical Garden of the University of Leipzig, Germany.

### 3.2. Isolation and Identification of Salicortin (**1**), HCH-Salicortin (**2**), Tremulacin (**3**) and Idescarpin (**4**)

Because of the very complex leaf matrix of Salicaceae plants, a solid-phase extraction (SPE) separation course for the isolation of salicinoids was developed [18]. Generally, the plant material was harvested, snap-frozen in liquid nitrogen and subjected to lyophylization. Subsequently, after the exhaustive extraction of the ground material with 70% methanol in water, the extract was pre-purified by SPE followed by chromatography on Sephadex LH-20. From the resulting fractions, salicinoids were purified using HPLC (see supplementary information).

To determine metabolite concentration, salicortin (**1**), HCH-salicortin (**2**) and tremulacin (**3**) were isolated from 454 mg lyophylized *Populus trichocarpa* × *deltoides* Beaupré leaf material. Accordingly, the material was extracted (6 × 30 mL) with MeOH/H_2_O (7:3 v/v), resulting in 201 mg crude extract after solvent evaporation. The remaining material was reconstituted with water and centrifuged to separate insoluble matter. The supernatant was subjected to pre-separation on HR-X SPE cartridges. After loading the cartridge, washing with H_2_O (6 mL) and elution with MeOH, the eluate was passed through a DPA-6S polyamide SPE cartridge for the removal of procyanidins. The filtrate was evaporated to dryness by vacuum centrifugation. After reconstitution with MeOH, column separation on Sephadex LH-20 (160 mm × 15 mm) with MeOH as eluent was performed. The volumes of fractions taken were as follows: 1–20 mL, 2–10 mL, 3–10 mL, 4–10 mL, 5–15 mL, and 6–20 mL. Fractions 2 and 3 contained the desired compounds. Both fractions were pooled and evaporated to dryness, giving 68 mg pre-purified material. Aliquots were subjected to HPLC in order to isolate the salicinoids. To prevent sample from decomposing during the evaporation of the acidic HPLC solvent, each fractionated compound was immobilized on an HR-X SPE. Final evaporation of the MeOH eluate gave the pure compounds. The amount of salicortin isolated was 2.70 mg per gram lyophylized leaf material (0.3%). HCH-salicortin and tremulacin were obtained in yields of 0.1% and 0.2%, respectively.

To isolate idescarpin (**4**), 5.74 g of lyophylized *Idesia polycarpa* leaf material was exhaustively (6 × 150 mL) extracted using MeOH/H_2_O (7:3 v/v), giving 1.6 g crude extract. This crude material was reconstituted with MeOH (70 mL), and the insoluble precipitate was removed by means of centrifugation. The combined supernatants were pooled and dried using a vacuum centrifuge. The dried extract was then reconstituted with water (70 mL), split into three equal portions and subjected to separation on HR-X SPE columns. After conditioning with MeOH and equilibration with H_2_O, the water extract was loaded and columns were subsequently washed with H_2_O (6 mL). Elution with MeOH and solvent evaporation by vacuum centrifugation gave 720 mg of pre-purified extract. A portion of 115 mg was then subjected to HPLC, resulting in 10 mg pure **4**. Accordingly, the leaf material used for the isolation contained about 1.1% of idescarpin (**4**), based on dry weight.

## 4. Conclusions

By comparing spectroscopic data of salicortin (**1**), HCH-salicortin (**2**) and tremulacin (**3**) with those of idescarpin (**4**), we conclude that the HCH moiety in all isolated salicinoids is (*S*)-configured at the stereogenic centers C-9 and C-2" (Figure 1). Moreover, one can assume that the HCH moiety in salicinoids is generally (*S*)-configured, since other compounds bearing this structure element have never been shown to represent diastereomeric mixtures. It is also unlikely that these compounds are biosynthesized through routes other than those of the examined salicinoids. An identical assumption was made based on the results of chemoenzymatic studies; however, without a direct comparison with authentic samples [19]. It has been shown that leaves of *Idesia polycarpa* contain high amounts of (−)-idescarpin (**4**) and it is suggested that **4** serves as a defensive compound against herbivores and fungal infection.

## Supplementary Materials

Supplementary materials can be accessed at: http://www.mdpi.com/1420-3049/20/04/5566/s1.
