# Supplementary material for: The Absolute Configuration of Salicortin, HCH-Salicortin and Tremulacin from Populus trichocarpa × deltoides Beaupré
Source: Molecules. 2015 Mar 30;20(4):5566–73. doi: 10.3390/molecules20045566 (PMC6272461; doi:10.3390/molecules20045566)
Supplement: Supplementary file 1 [file molecules-20-05566-s001.pdf]

Supplementary Information

S1.1: HPLC method- salicinoids from *Populus trichocarpa* × *deltoides* Beaupré
S1.2: HPLC method- salicinoids from *Idesia polycarpa*
S1.3: Specific optical rotation of salicortin (**1**) and idescarpin (**4**)

S2.1: Salicortin (**1**), ^{1}H-NMR spectrum (500 MHz, MeCN-d_3)
S2.2: Salicortin (**1**), ^{13}C-NMR spectrum (125 MHz, MeCN-d_3)
S2.3: Salicortin (**1**), ^{1}H-^{1}H COSY spectrum (500 MHz, MeCN-d_3)
S2.4: Salicortin (**1**), ^{1}H-^{13}C HSQC spectrum (500 MHz, MeCN-d_3)
S2.5: Salicortin (**1**), ^{1}H-^{13}C HMBC spectrum (500 MHz, MeCN-d_3)
S2.6: Salicortin (**1**), structure with chemical shifts (MeCN-d_3)
S2.7: Salicortin (**1**), result of the HRMS measurement
S2.8: Salicortin (**1**), results of the CD measurement

S3.1: Tremulacin (**2**), ^{1}H-NMR spectrum (700 MHz, MeCN-d_3)
S3.2: Tremulacin (**2**), ^{1}H-^{1}H COSY spectrum (700 MHz, MeCN-d_3)
S3.3: Tremulacin (**2**), ^{1}H-^{13}C HSQC spectrum (700 MHz, MeCN-d_3)
S3.4: Tremulacin (**2**), ^{1}H-^{13}C HMBC spectrum (700 MHz, MeCN-d_3)
S3.5: Tremulacin (**2**), structure with chemical shifts (MeCN-d_3)
S3.6: Tremulacin (**2**), result of the HRMS measurement
S3.7: Tremulacin (**2**), results of the CD measurement

S4.1: HCH-Salicortin (**3**), ^{1}H-NMR spectrum (700 MHz, MeCN-d_3)
S4.2: HCH-Salicortin (**3**), ^{1}H-^{1}H COSY spectrum (700 MHz, MeCN-d_3)
S4.3: HCH-Salicortin (**3**), ^{1}H-^{13}C HSQC spectrum (700 MHz, MeCN-d_3)
S4.4: HCH-Salicortin (**3**), ^{1}H-^{13}C HMBC spectrum (700 MHz, MeCN-d_3)
S4.5: HCH-Salicortin (**3**), structure with chemical shifts (MeCN-d_3)
S4.6: HCH-Salicortin (**3**), result of the HRMS measurement
S4.7: HCH-Salicortin (**3**), results of the CD measurement

S5.1: Idescarpin (**4**), ^{1}H-NMR spectrum (500 MHz, MeCN-d_3)
S5.2: Idescarpin (**4**), ^{13}C NMR spectrum (125 MHz, MeCN-d_3)
S5.3: Idescarpin (**4**), ^{1}H-^{1}H COSY spectrum (500 MHz, MeCN-d_3)
S5.4: Idescarpin (**4**), ^{1}H-^{13}C HSQC spectrum (500 MHz, MeCN-d_3)
S5.5: Idescarpin (**4**), ^{1}H-^{13}C HMBC spectrum (500 MHz, MeCN-d_3)
S5.6: Idescarpin (**4**), structure with chemical shifts (MeCN-d_3)
S5.7: Idescarpin (**4**), result of the HRMS measurement
S5.8: Idescarpin (**4**), results of the CD measurement

Table S1.1. HPLC method- salicinoids from *Populus trichocarpa* × *deltoides* Beaupré.

**HPLC Method for Isolation of Salicinoids (1–3) (Sample Concentration 3 mg/mL)**			
**Column Temp.**	**35 °C**	**Injection Volume**	**40 µL**
**Flow Rate**	**0.8 mL/min**	***Isis Nucleodur 250 mm × 4.6 mm; 5 µm; MN***	

**Time t [min]**	**Solvent A H_2O (0.1% FA) in %**	**Solvent B MeOH (0.1% FA) in %**
0	100	0
5	100	0
10	85	15
35	70	30
85	50	50
90	0	100
100	0	100
110	100	0
115	100	0

Retention times: salicortin (**1**): 42.6 min; HCH-salicortin (**2**): 63.6 min; tremulacin (**3**): 87.8 min.

Table S1.2. HPLC method- salicinoids from *Idesia polycarpa*.

**HPLC Method for Isolation of Idescarpin (4) (Sample Concentration 115 mg/mL)**			
**Column Temp.**	**40 °C**	**Injection Volume**	**5 µL**
**Flow Rate**	**0.8 mL/min**	***Isis Nucleodur 250 mm × 4.6 mm; 5 µm; MN***	

**Time t [min]**	**Solvent A H_2O (0.1% FA) in %**	**Solvent B MeOH (0.1% FA) in %**
0	67.5	32.5
1	67.5	32.5
21	32	68
25	0	100
30	0	100
35	67.5	32.5
40	67.5	32.5

Retention times: idescarpin (**4**): 21.7 min.

Table S1.3. Specific optical rotation of salicortin (**1**) and idescarpin (**4**).

Measurement	Salicortin (1)		Idescarpin (4)
	$[\alpha]_D^{22}$ (c 0.72; MeOH)	$[\alpha]_D^{22}$ (c 0.65; H_2O)	$[\alpha]_D^{22}$ (c 0.73; MeOH)
1	−123.91°	−119.06°	−57.12°
2	−123.67°	−118.84°	−56.97°
3	−124.06°	−118.55°	−57.59°
4	−124.06°	−118.83°	−57.44°
5	−124.10°	−118.87°	−57.66°
6	−123.53°	−118.22°	−56.90°
7	−124.18°	−118.43°	−57.48°
8	−123.95°	−118.98°	−57.40°
9	−123.87°	−118.42°	−57.60°
10	−123.62°	−118.06°	−57.27°
mean $[\alpha]_D$	−123.89°	−118.63°	−57.34°
stdev.	±0.22°	±0.34°	±0.27°

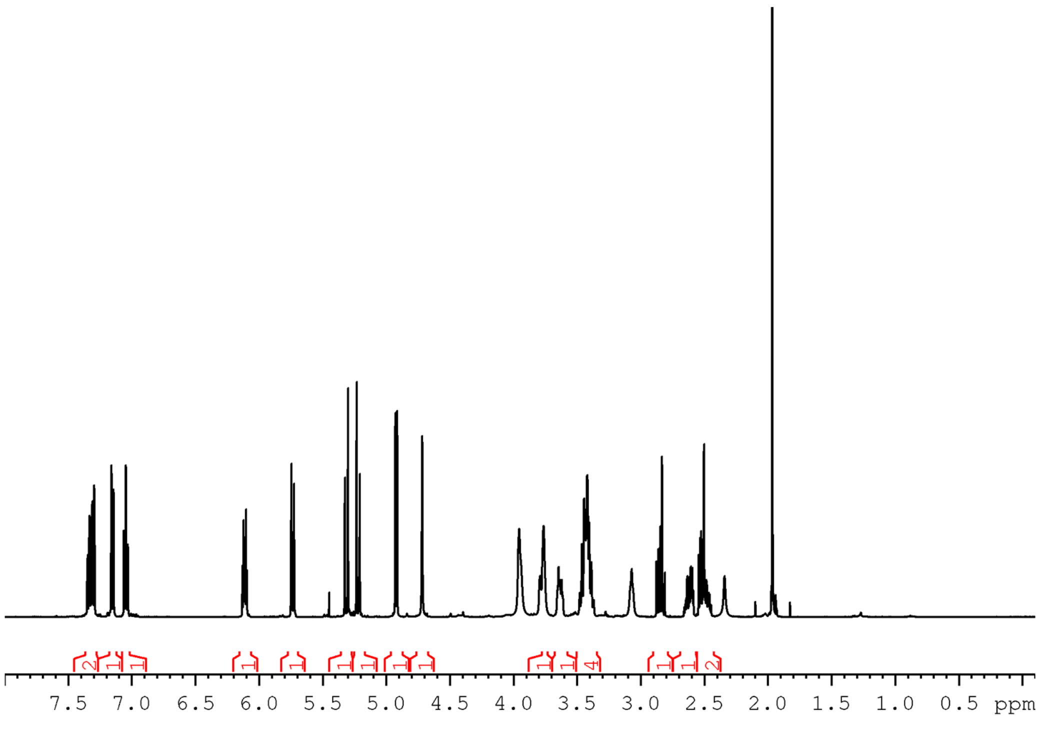

Figure S2.1. Salicortin (**1**), ^{1}H-NMR spectrum (500 MHz, MeCN-d_3).

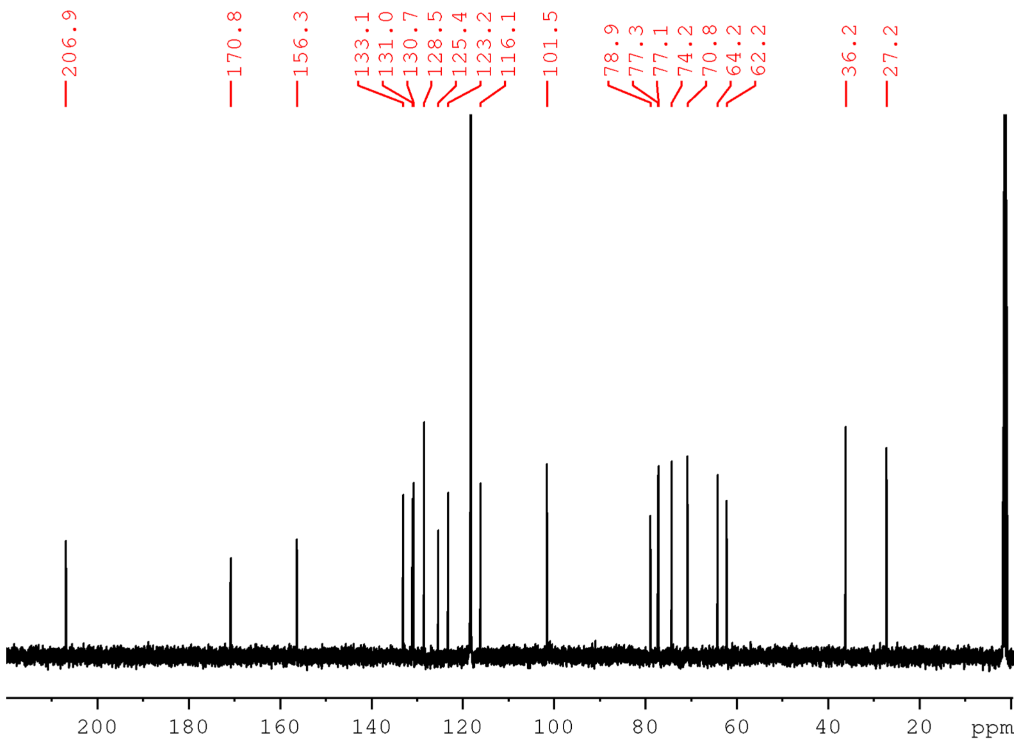

Figure S2.2. Salicortin (**1**), ^{13}C-NMR spectrum (125 MHz, MeCN-d_3).

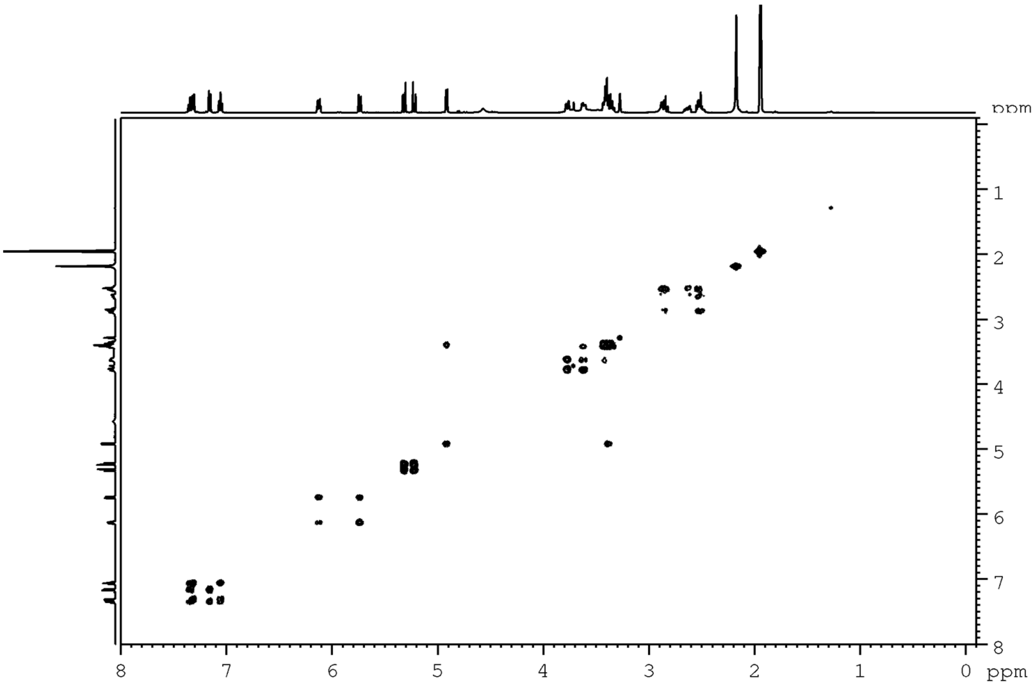

Figure S2.3. Salicortin (**1**), ^{1}H-^{1}H COSY spectrum (500 MHz, MeCN-d_3).

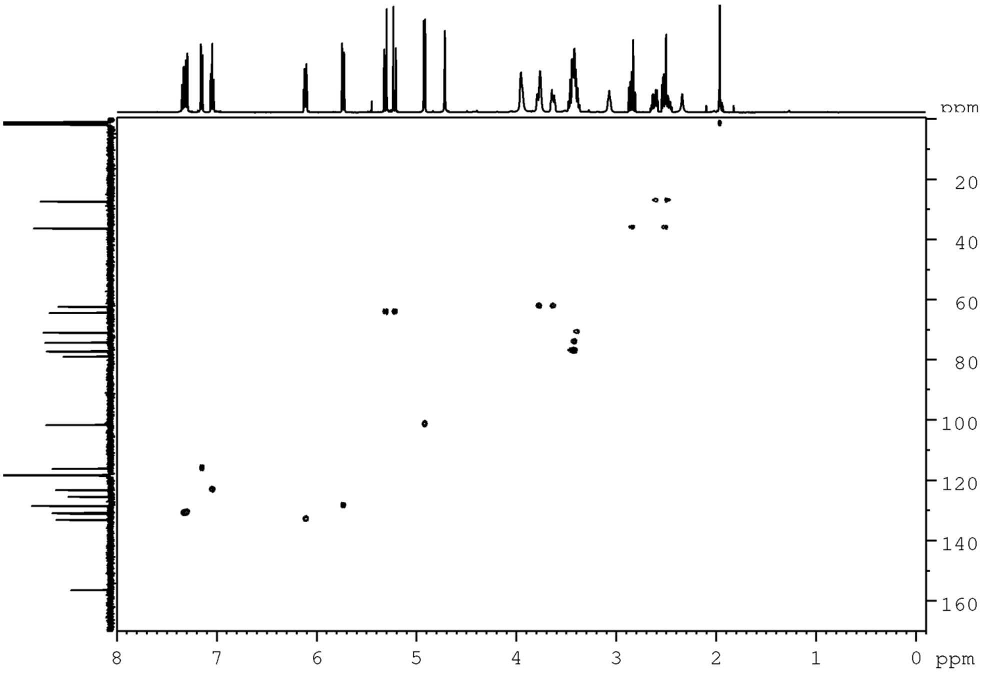

Figure S2.4. Salicortin (**1**), ^{1}H-^{13}C HSQC spectrum (500 MHz, MeCN-*d*3).

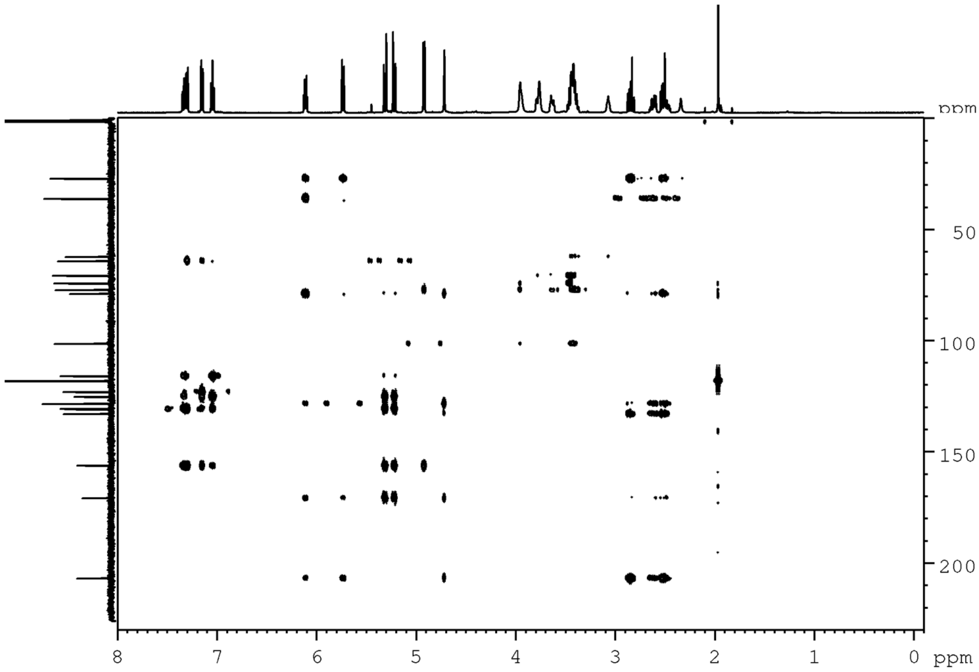

Figure S2.5. Salicortin (**1**), ^{1}H-^{13}C HMBC spectrum (500 MHz, MeCN-*d*3).

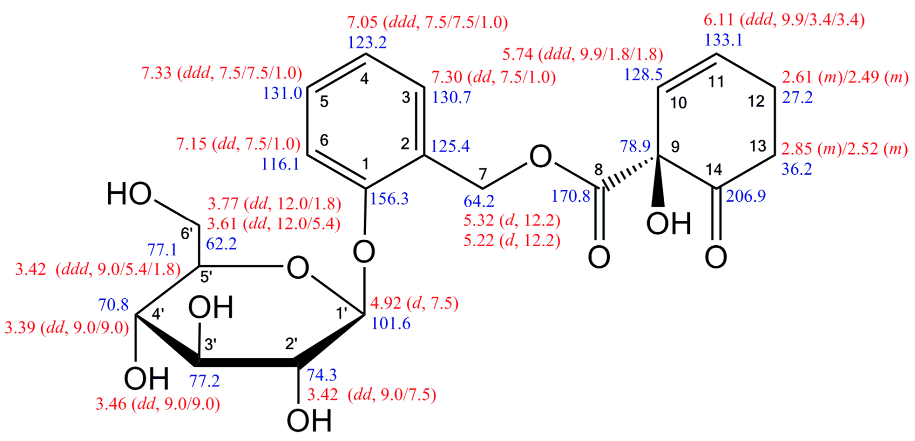

Figure S2.6. Salicortin (**1**), structure with chemical shifts (MeCN-d_3), multiplicities and coupling constants (J in Hz). Red: ^{1}H-NMR (500 MHz); blue: ^{13}C-NMR (125 MHz).

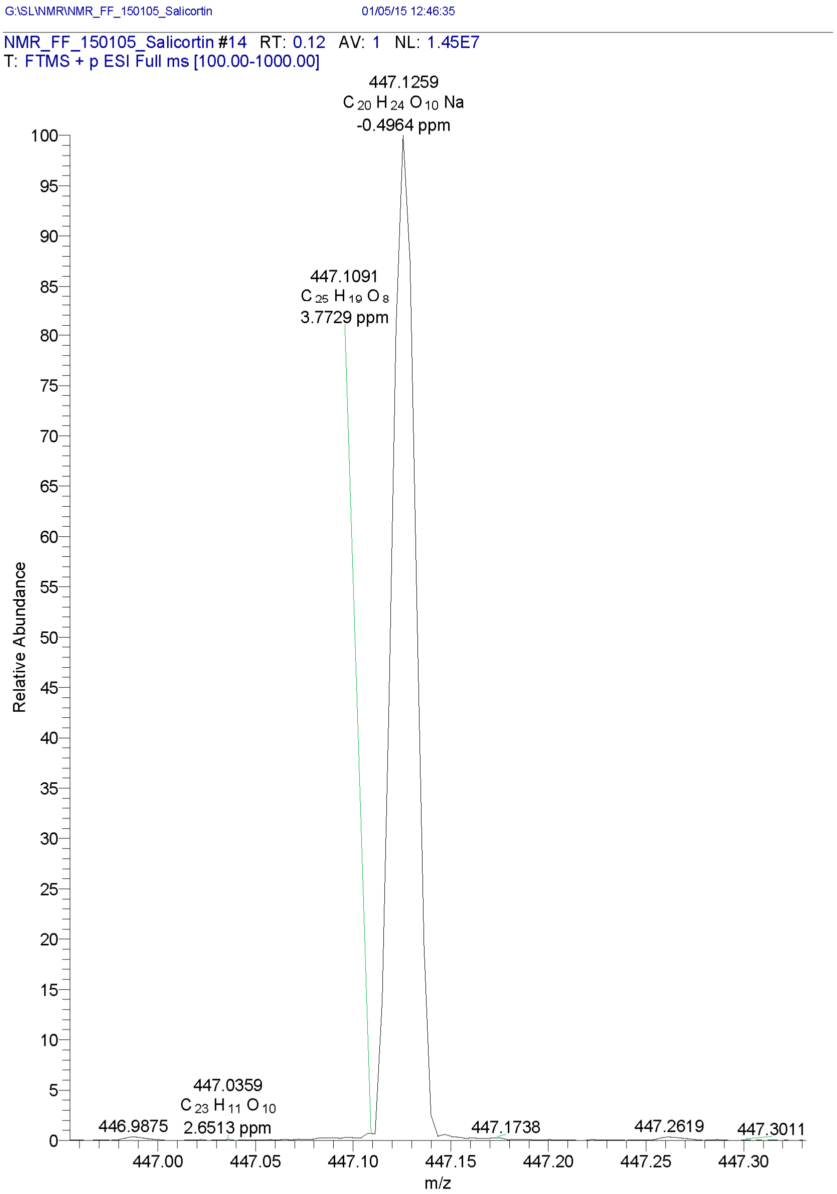

Figure S2.7. Salicortin (**1**), result of the HRMS measurement.

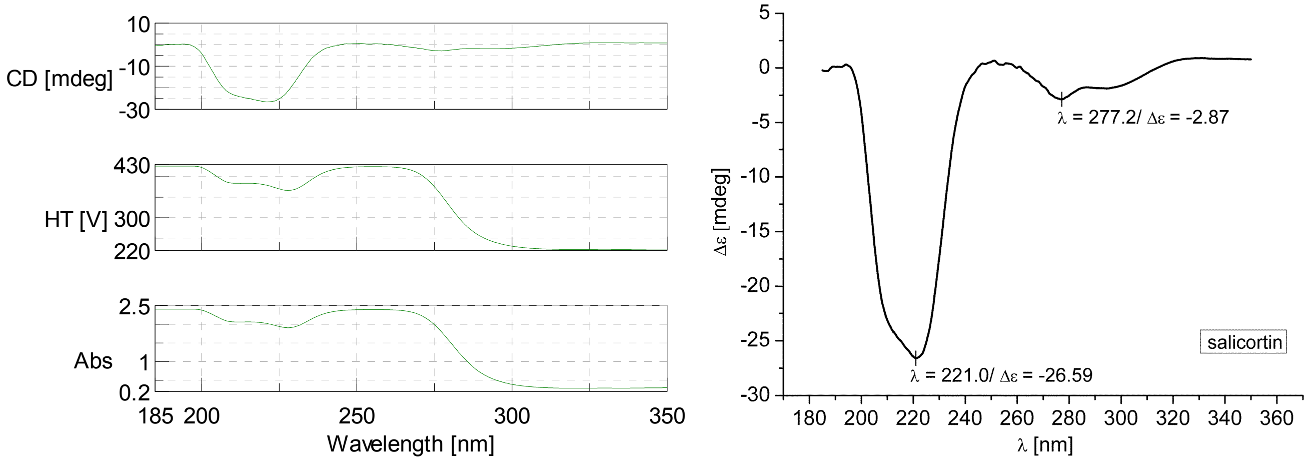

(a) (b)

Figure S2.8. Salicortin (**1**), (**a**) results of the CD measurement (concentration 0.70 mg/mL (1.66 mM in MeOH), cuvette width 1 mm). (**b**) Molar circular dichroism Δε at maximum wavelengths.

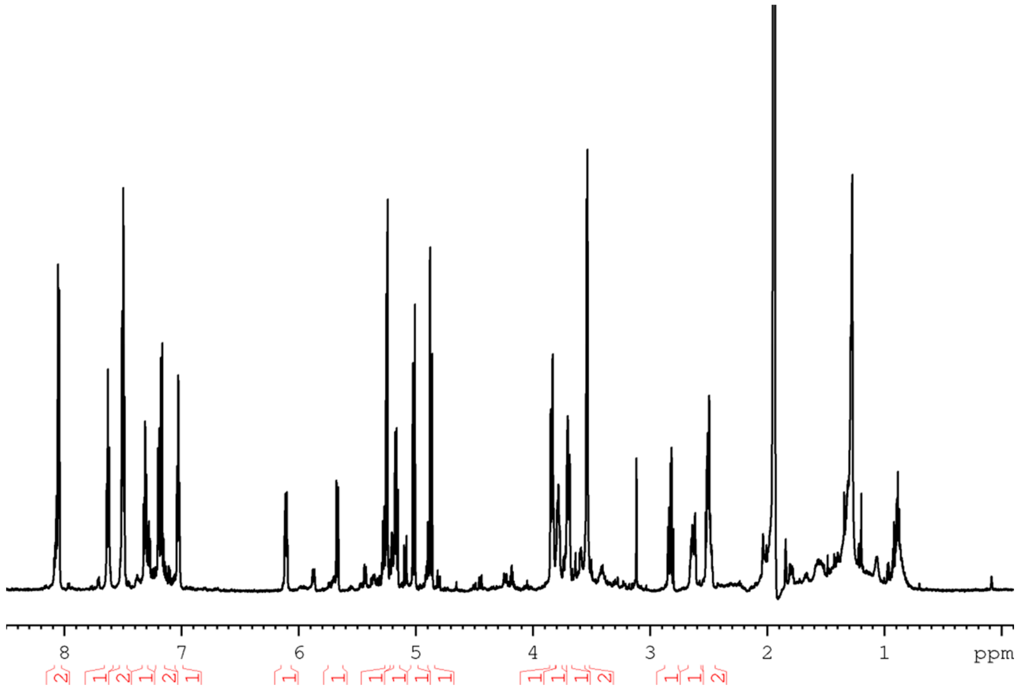

Figure S3.1. Tremulacin (**2**), ^{1}H-NMR spectrum (700 MHz, MeCN-d_3).

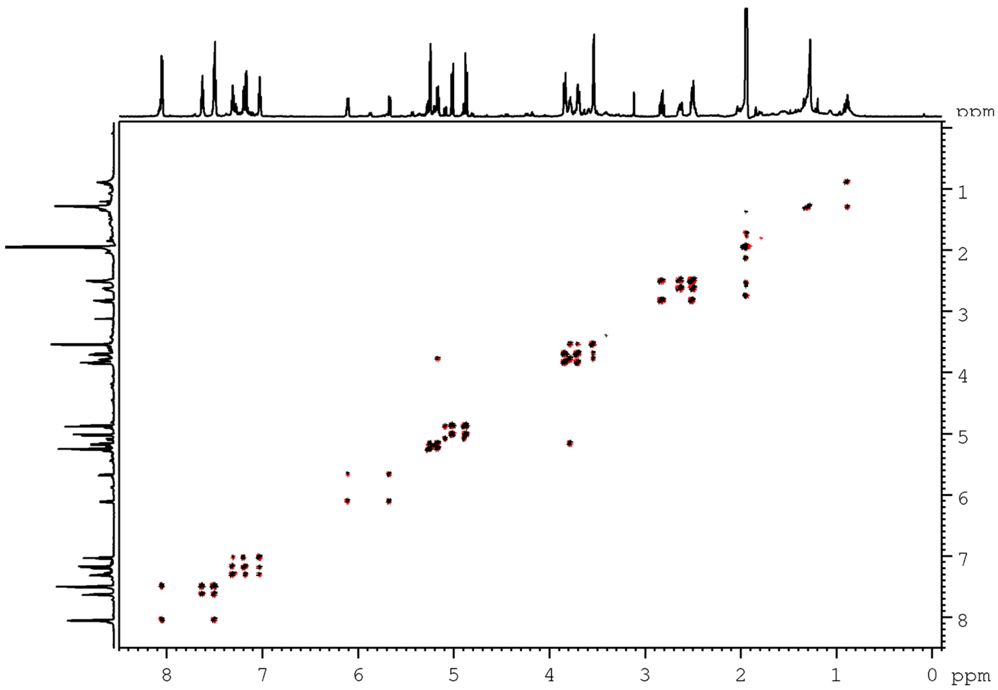

Figure S3.2. Tremulacin (**2**), ^{1}H-^{1}H COSY spectrum (700 MHz, MeCN-*d*3).

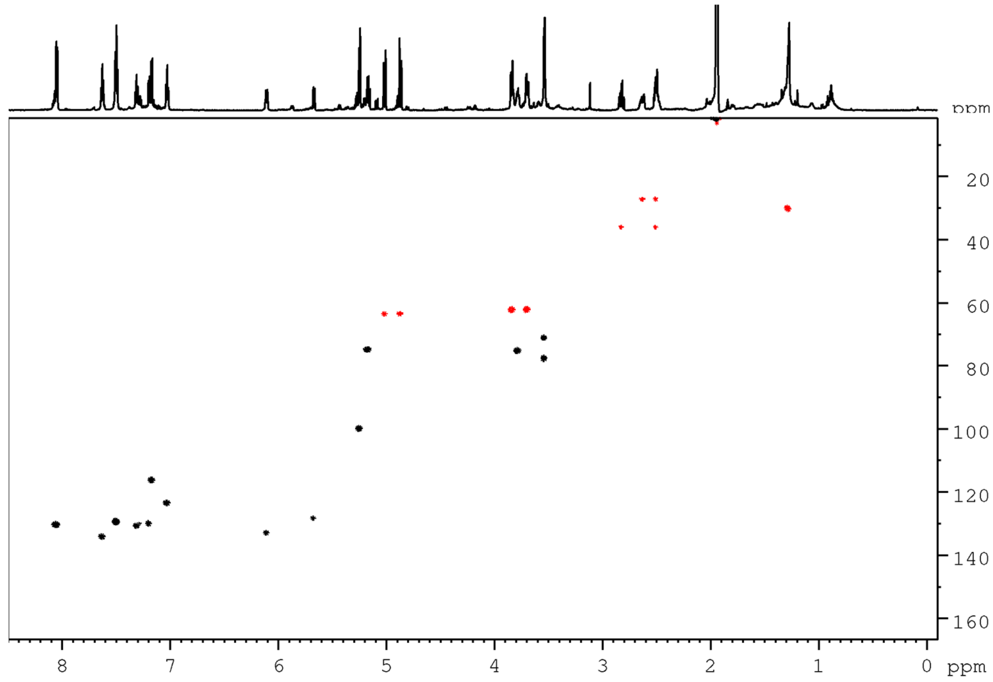

Figure S3.3. Tremulacin (**2**), ^{1}H-^{13}C HSQC spectrum (700 MHz, MeCN-*d*3).

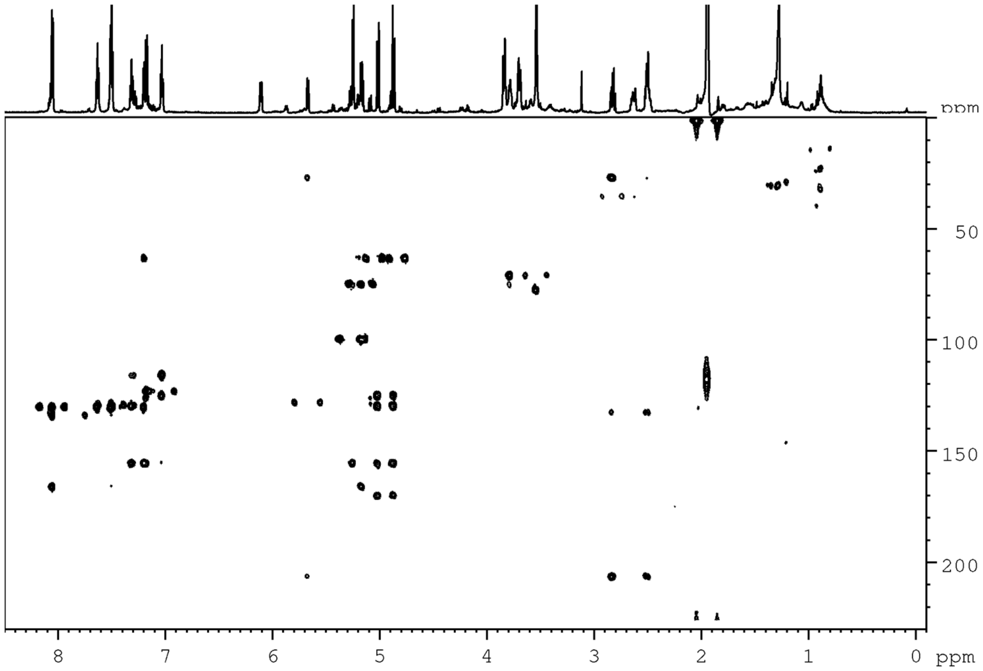

Figure S3.4. Tremulacin (**2**), ^{1}H-^{13}C HMBC spectrum (700 MHz, MeCN-d_3).

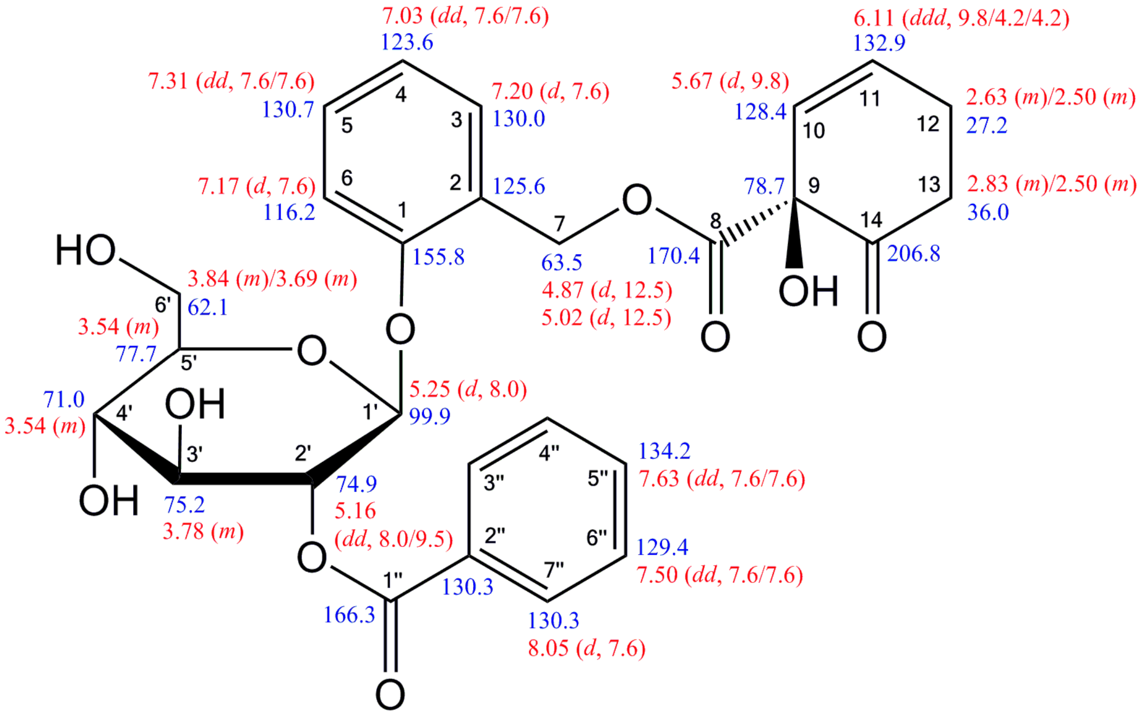

Figure S3.5. Tremulacin (**2**), structure with chemical shifts (MeCN-d_3), multiplicities and coupling constants (*J* in Hz). Red: ^{1}H-NMR (700 MHz); blue: ^{13}C-NMR (175 MHz).

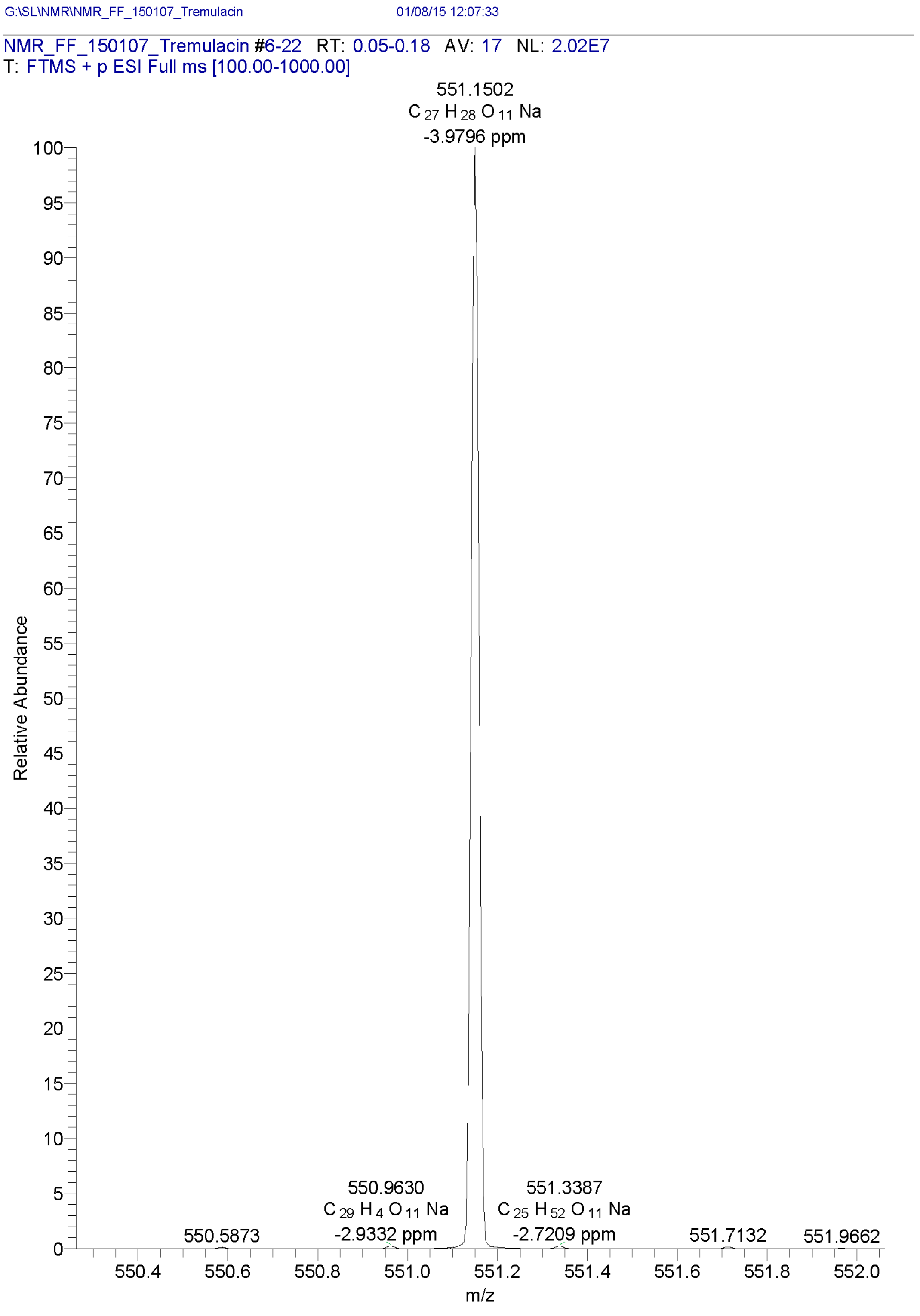

Figure S3.6. Tremulacin (**2**), result of the HRMS measurement.

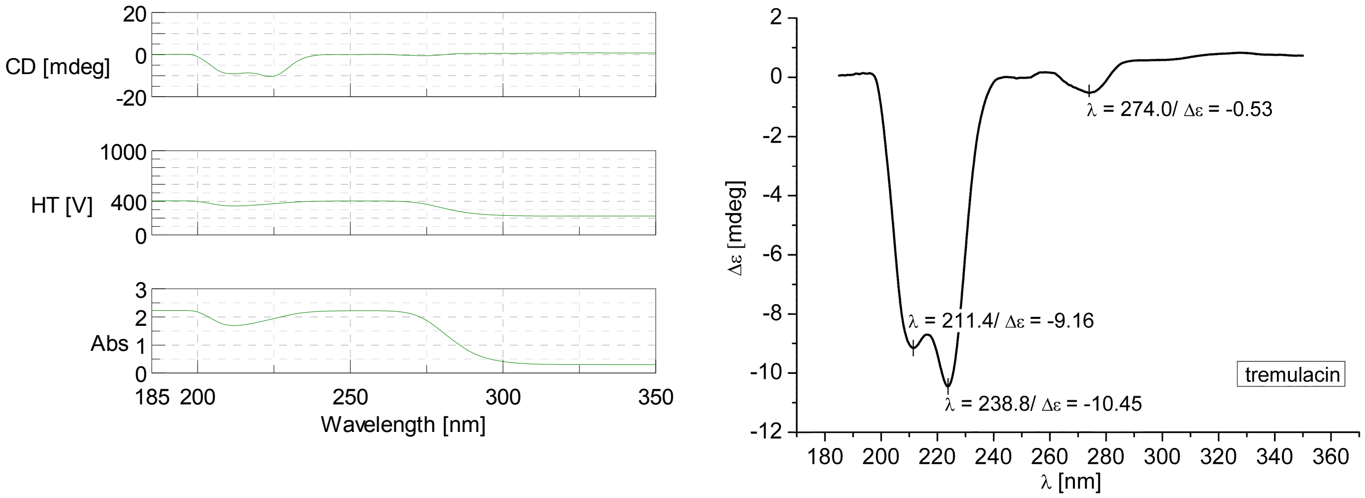

(a) (b)

Figure S3.7. Tremulacin (**2**), (**a**) results of the CD measurement (concentration 0.76 mg/mL (1.44 mM in MeOH), cuvette width 1 mm). (**b**) Molar circular dichroism Δε at maximum wavelengths.

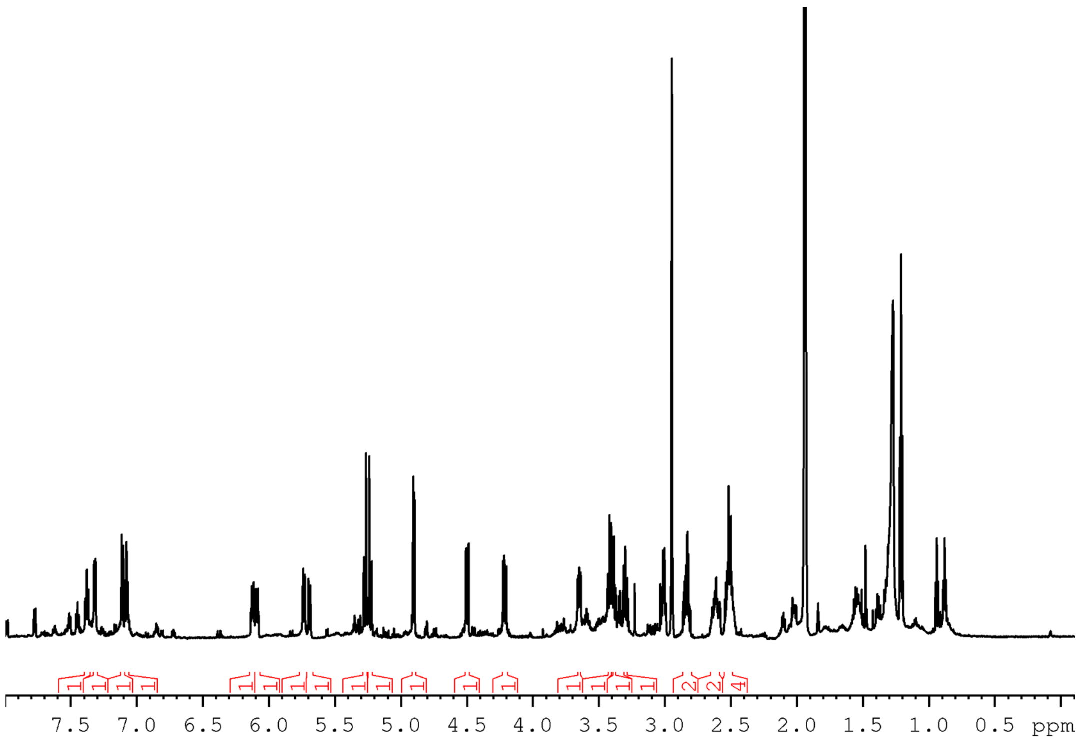

Figure S4.1. HCH-Salicortin (**3**), ^{1}H-NMR spectrum (700 MHz, MeCN-d_3).

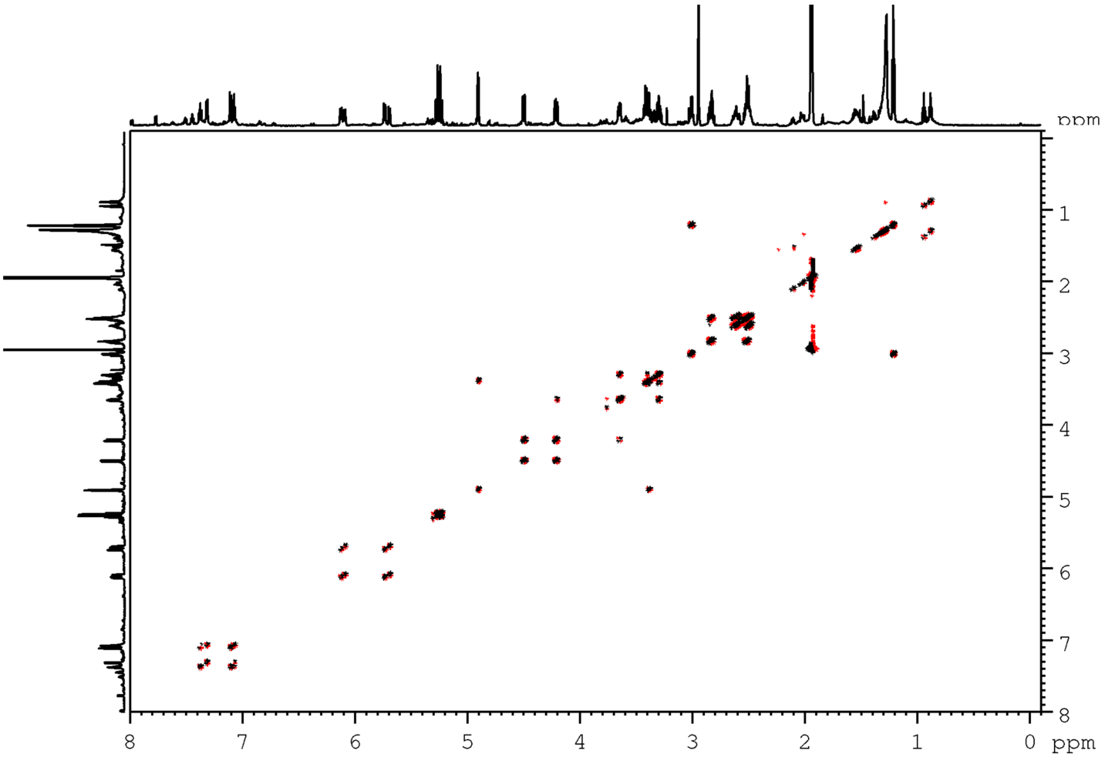

Figure S4.2. HCH-Salicortin (**3**), ^{1}H-^{1}H COSY spectrum (700 MHz, MeCN-d_3).

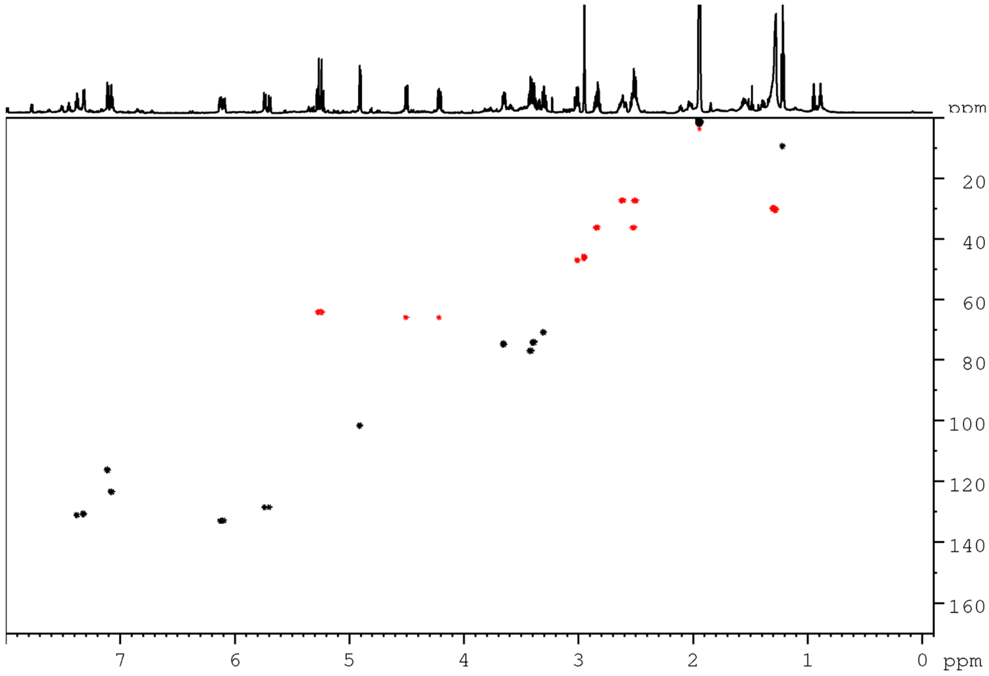

Figure S4.3. HCH-Salicortin (**3**), ^{1}H-^{13}C HSQC spectrum (700 MHz, MeCN-d_3).

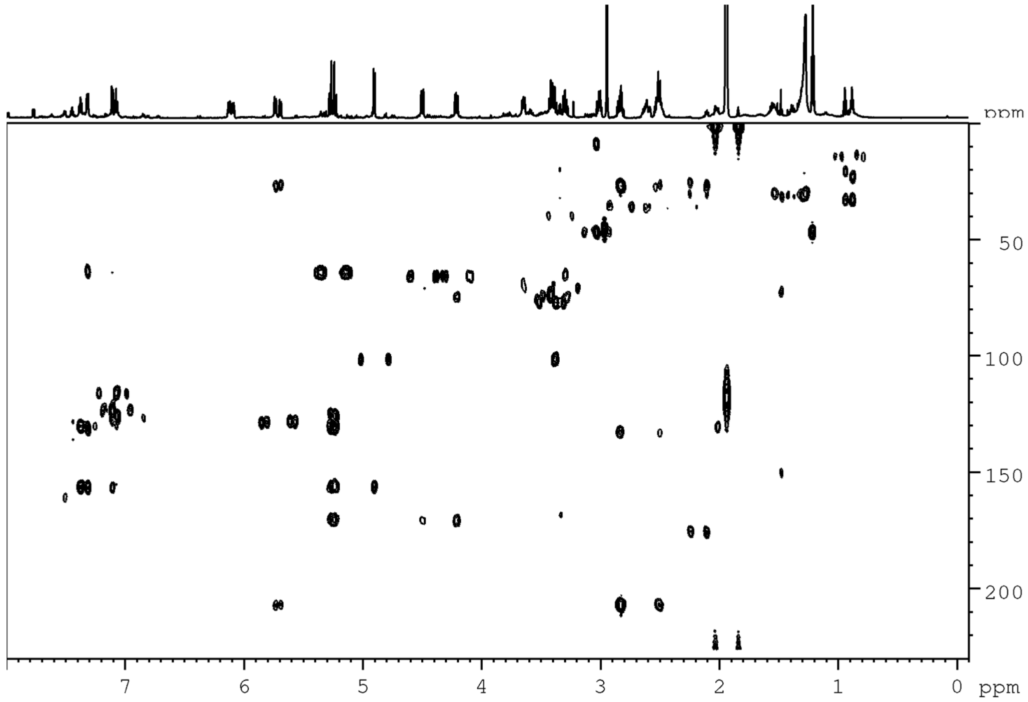

Figure S4.4. HCH-Salicortin (**3**), ^{1}H-^{13}C HMBC spectrum (700 MHz, MeCN-*d*3).

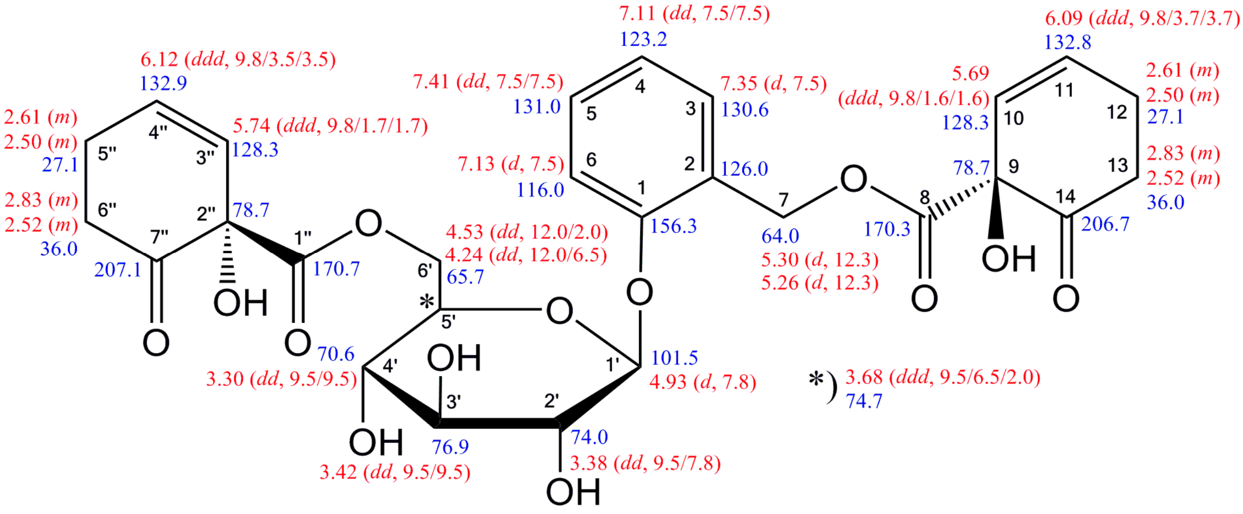

Figure S4.5. HCH-Salicortin (**3**), structure with chemical shifts (MeCN-*d*3) multiplicities and coupling constants (*J* in Hz). Red: ^{1}H-NMR (700 MHz); blue: ^{13}C-NMR (175 MHz).

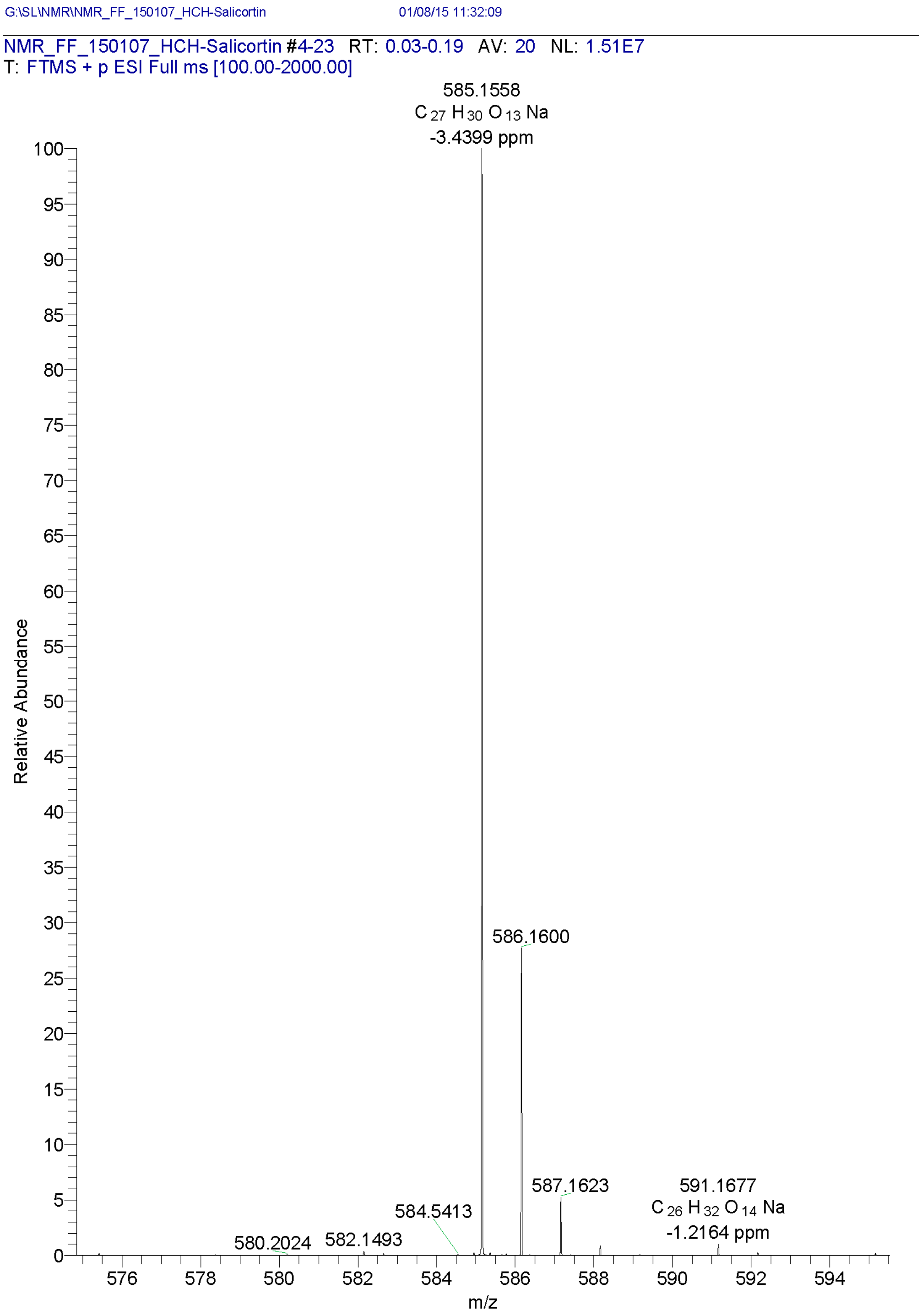

Figure S4.6. HCH-Salicortin (**3**), result of the HRMS measurement.

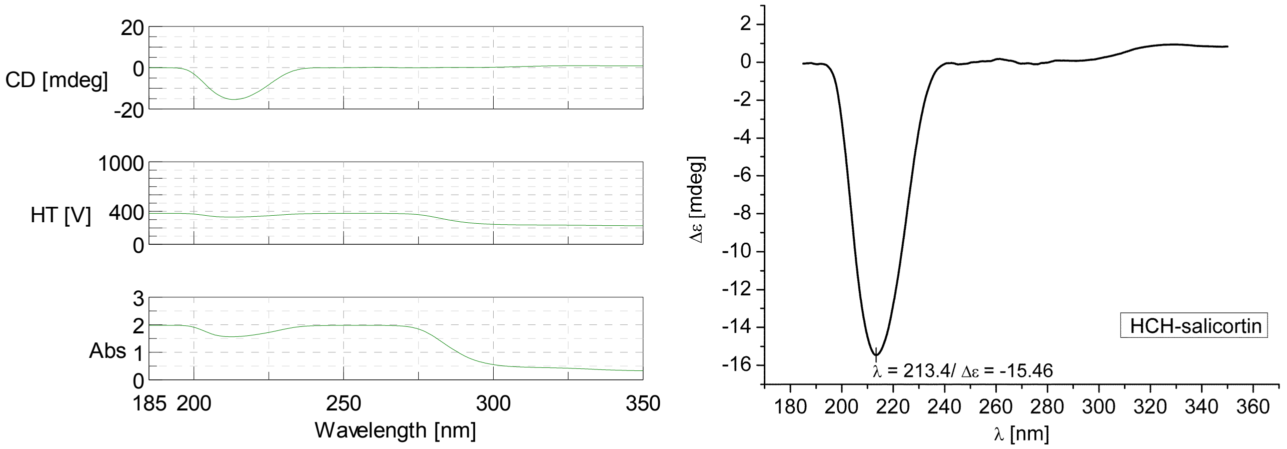

(**a**) (**b**)

Figure S4.7. HCH-Salicortin (**3**), (**a**) results of the CD measurement (concentration 0.67 mg/mL (1.19 mM in MeOH), cuvette width 1 mm). (**b**) Molar circular dichroism Δε at maximum wavelength.

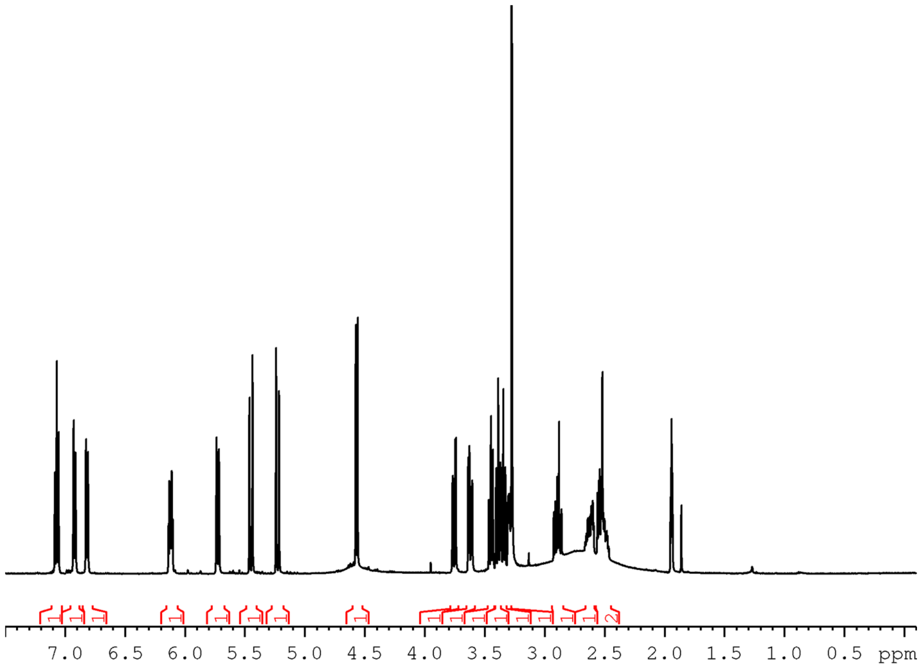

Figure S5.1. Idescarpin (**4**), ^{1}H-NMR spectrum (500 MHz, MeCN-d_3).

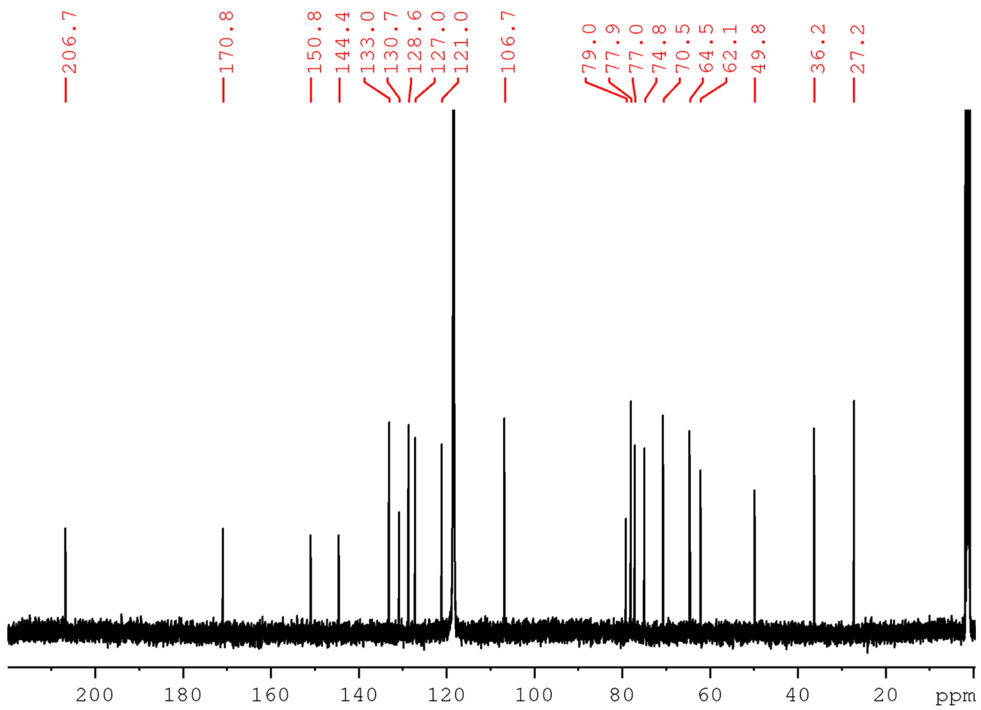

Figure S5.2. Idescarpin (**4**), ^{13}C-NMR spectrum (125 MHz, MeCN-d_3).

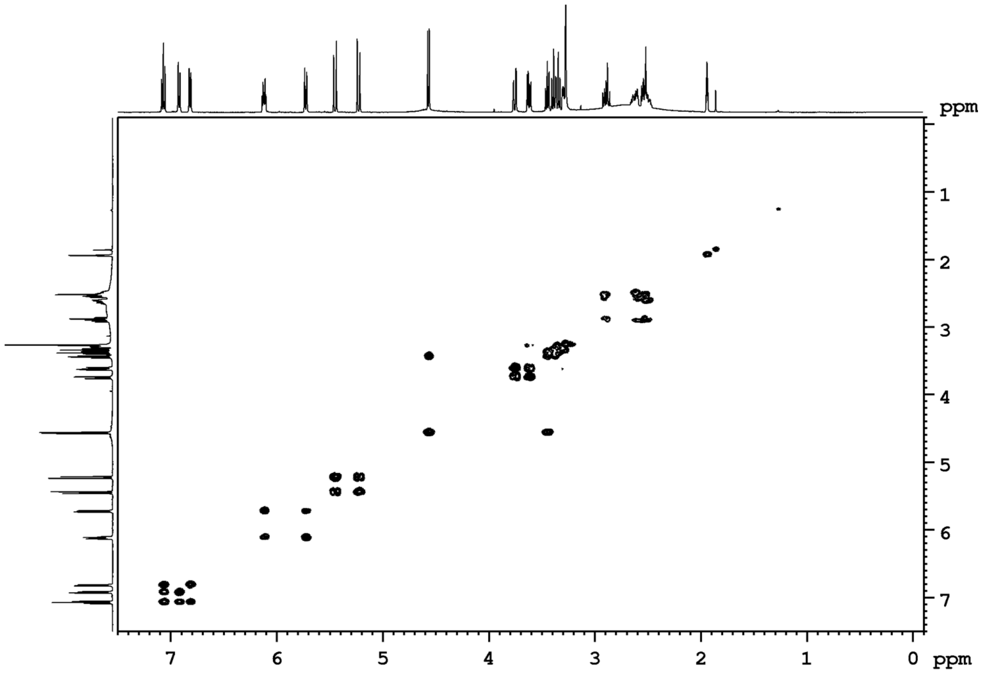

Figure S5.3. Idescarpin (**4**), ^{1}H-^{1}H COSY spectrum (500 MHz, MeCN-d_3).

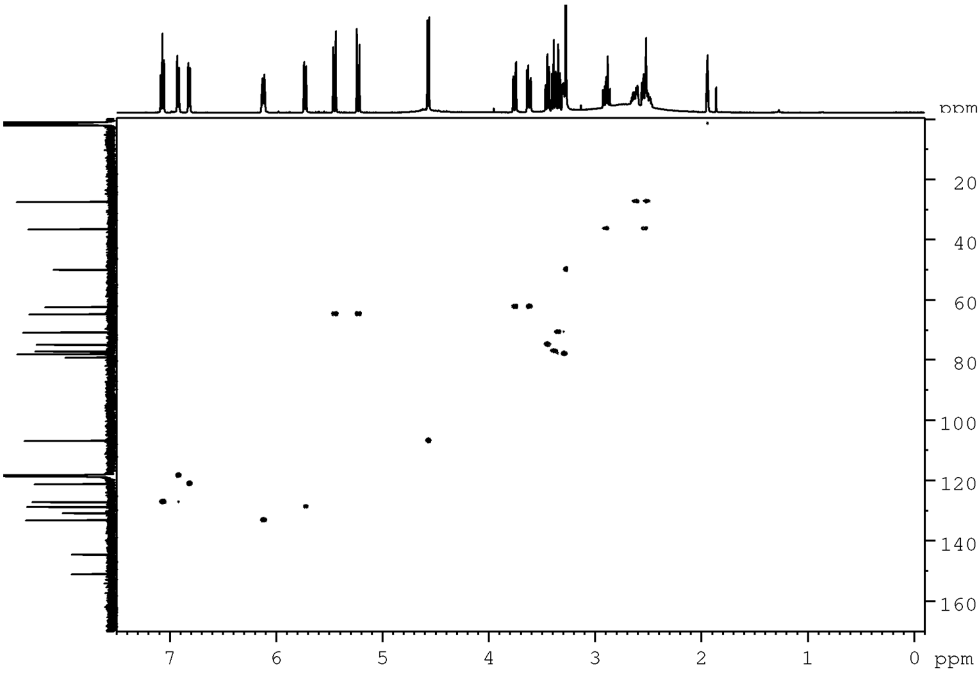

Figure S5.4. Idescarpin (**4**), ^{1}H-^{13}C HSQC spectrum (500 MHz, MeCN-*d*3).

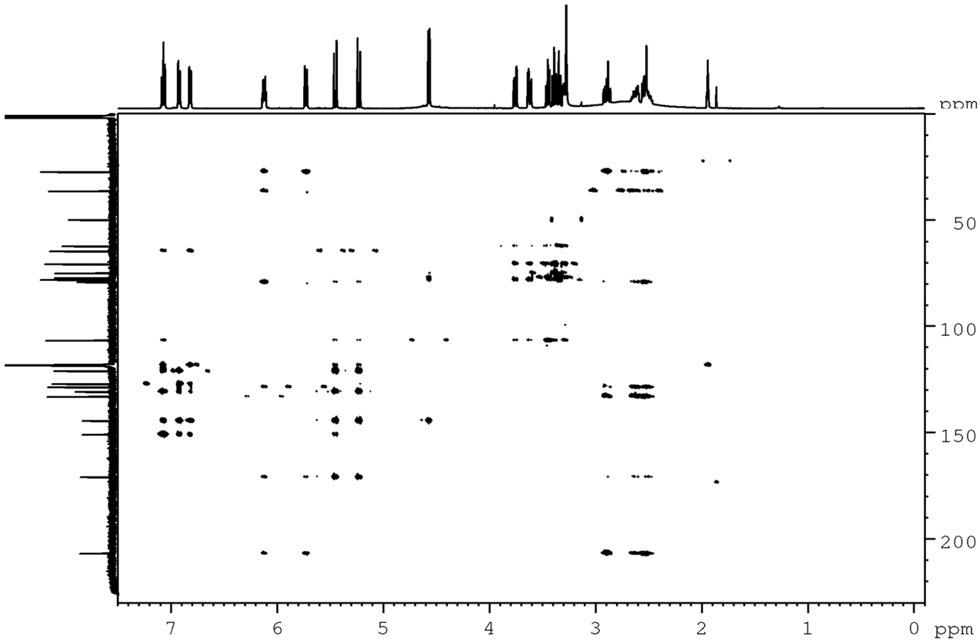

Figure S5.5. Idescarpin (**4**), ^{1}H-^{13}C HMBC spectrum (500 MHz, MeCN-*d*3).

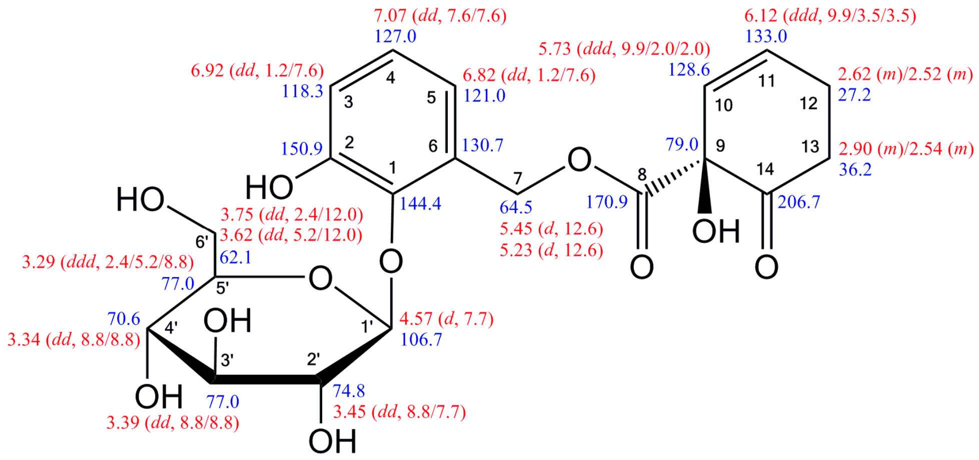

Figure S5.6. Idescarpin (**4**), structure with chemical shifts (MeCN-d_3), multiplicities and coupling constants (J in Hz). Red: ^{1}H-NMR (500 MHz); blue: ^{13}C-NMR (125 MHz).

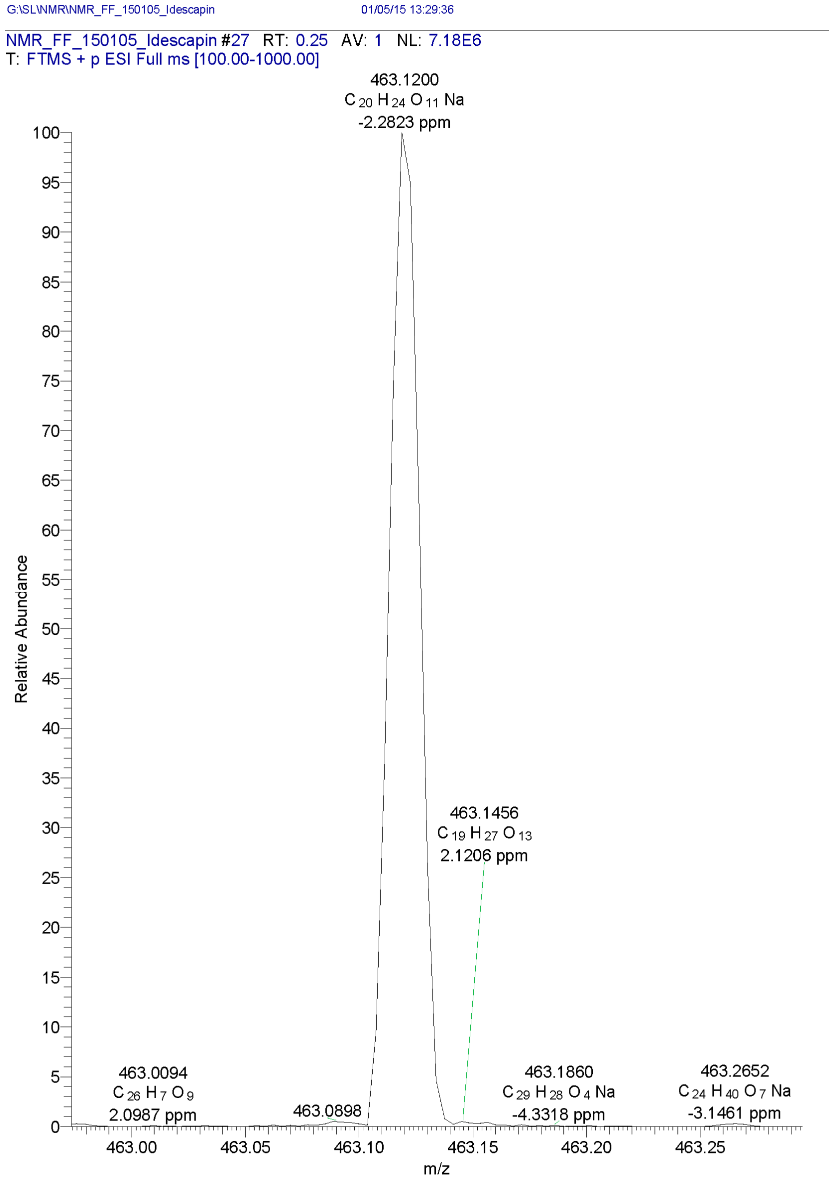

Figure S5.7. Idescarpin (**4**), result of the HRMS measurement.

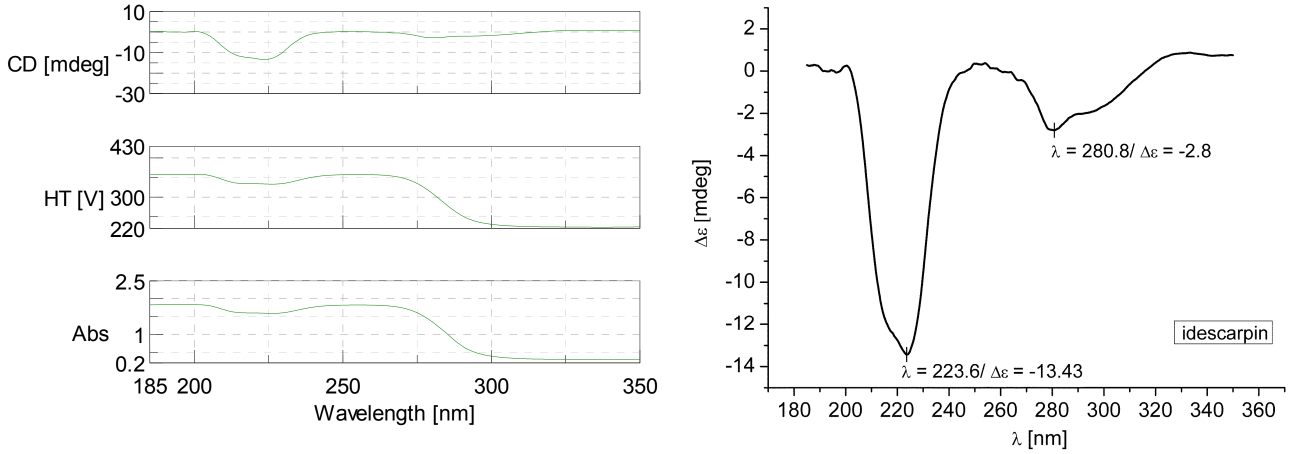

(**a**) (**b**)

Figure S5.8. Idescarpin (**4**), (**a**) results of the CD measurement (concentration 0.71 mg/mL (1.61 mM in MeOH), cuvette width 1 mm). (**b**) Molar circular dichroism Δε at maximum wavelengths.
